# Chikungunya in a pediatric cohort: Asymptomatic infection, seroconversion, and chronicity rates

**DOI:** 10.1371/journal.pntd.0013254

**Published:** 2025-07-16

**Authors:** Blenda de Jesus Pereira, Michelle Queiroz Aguiar Brasil, Jessica de Jesus Silva, Juqueline Rocha Cristal, Isabele de Pádua Carvalho, Maria Carolina Prado Miranda, Daniela Paixão, Ricardo Khouri, Thiago Cerqueira-Silva, Fernanda Castro Boulos, Manoel Barral-Netto, Esper Georges Kalas, Aldina Barral, Antônio Bandeira, Viviane Sampaio Boaventura

**Affiliations:** 1 Laboratório de Medicina e Saúde Pública de Precisão, Instituto Gonçalo Moniz, Fundação Oswaldo Cruz, Salvador, Bahia, Brazil; 2 Universidade Federal da Bahia, Salvador, Bahia, Brazil; 3 Faculty of Epidemiology and Population Health, London School of Hygiene & Tropical Medicine, London, United Kingdom; 4 Instituto Butantan, São Paulo, São Paulo, Brazil; 5 Instituto de Investigação em Imunologia, São Paulo, Brazil; 6 Hospital das Clínicas da Faculdade de Medicina da Universidade de São Paulo São Paulo, Brazil; 7 Faculdade ZARNS, Salvador, Bahia, Brazil; 8 Laboratório Central (LACEN) da Bahia, Salvador, Bahia, Brazil; Florida Department of Health, UNITED STATES OF AMERICA

## Abstract

Chikungunya disease, caused by the chikungunya virus (CHIKV), is an acute febrile syndrome that frequently leads to chronic musculoskeletal manifestations. Little is known about the incidence, asymptomatic rate, seroconversion and chronicity after acute CHIKV infection in children and adolescents. We leveraged a nested cohort study within a phase III clinical trial of the Dengue vaccine by the Butantan Institute (DEN-03-IB), in Simões Filho (Bahia-Brazil) to characterize the dynamics of CHIKV infection in the pediatric population. 348 volunteers were included between 2018–2020 and followed for up to three years. Arbovirus surveillance was conducted during medical visits using 1) routine study visits with periodic blood collection; 2) visits due to adverse events (any symptom or illness); and 3) visits due to febrile episodes, with routine blood samples tested for chikungunya, Dengue, and Zika by viral RNA detection using RT-PCR. For cases with suspected arbovirus infection, symptoms and signs were collected with a structured questionnaire. At baseline, 7% (23/348) were positive for antichikungunya IgG. Among 311 that completed follow up (41 months, IQR 38–43), 17% tested positive for CHIKV, with 25 cases positive by RT-PCR and 28 cases by serology. 9.4% were asymptomatic and 3 (12%) developed chronic arthralgia. By the end of the study, only onefifth have been exposed to CHIKV despite several local outbreaks. Seroconversion rate among RT-PCR positive cases was 84%. Chronic arthralgia, though infrequent, was observed in the pediatric population. Our study demonstrates that, within the pediatric population, most CHIKV infections are symptomatic. We observed a small but significant frequency of negative antibody responses following acute infection and instances of chronic disease. These findings underscore the necessity for continuous surveillance and tailored interventions to tackle the unique challenges chikungunya presents in children and adolescents.

## Author summary

Chikungunya is an infectious disease that can frequently cause chronic and debilitating muscle skeletal symptoms. While adults often experience severe symptoms and long-term effects, less information is available on children. This study investigates chikungunya in children and adolescents, focusing on its occurrence in an endemic area, frequency of asymptomatic cases, seroconversion and chronic manifestations. By examining these aspects, our research provides critical insights into the disease in a young population, which is essential to improve public health strategies and planning effective interventions and mitigate its impact.

## Introduction

Over the past two decades, chikungunya, a mosquito-borne disease, has become a significant global public health issue [1]. The disease presents dengue-like symptoms, including high fever, severe joint pain, and rashes. Notably, up to 60% of those infected can progress to chronic conditions such as persistent arthralgia [1–3], significantly reducing their quality of life. With the ongoing effects of climate change and global warming, there is growing concern about the potential expansion of mosquito habitats, which could facilitate a wider spread of chikungunya and an increase in the prevalence of chronic symptoms [4,5]. Although commonly reported in adults, chikungunya virus (CHIKV) infection can also affect children and adolescents. Long-term surveillance studies in children are scarce and suggest that the infection tends to be asymptomatic and less frequently evolving to chronic disease [6–8]. Key aspects such as the frequency of asymptomatic infections, the rate of chronicity, and seroconversion after acute CHIKV infection remain poorly understood. Accurately determining the incidence of CHIKV infections is critical to comprehend the magnitude of the problem in the pediatric population. Identifying asymptomatic cases is crucial for elucidating virus transmission dynamics and formulating effective control measures. Moreover, understanding the seroconversion rate can provide valuable insights regarding the number of individuals who remain susceptible after infection and aid in planning vaccination strategies.

This study aimed to assess the incidence, clinical presentation, seroconversion, and chronicity of chikungunya virus (CHIKV) infection in children and adolescents. Over four years, we monitored 348 participants aged 2–17 within a clinical trial evaluating the efficacy and safety of the Butantan-DV dengue vaccine. As part of an active arbovirus surveillance plan, structured queries and molecular biology tests were used to detect acute infections with CHIKV, Dengue (DENV), and Zika (ZIKV) viruses. Chikungunya cases were then analyzed to provide insights into disease burden and duration of immune response following infection.

## Methods

### Research ethics

The study was approved by the Research Ethics Committee of Gonçalo Moniz Institute (Approval number 3,778,546 - CAAE 18918519.6.0000.0040). Written informed consent was obtained from both parents or legal guardians. For participants aged 12–17 years, a written free and informed assent form was also obtained. As the study protocol granted access to the original databases for supplementary arbovirus research, no additional consent was required for this study.

### Study design and population sample

This nested cohort sub-study is part of the phase 3 randomized controlled trial (RCT) evaluating the Butantan-Dengue Vaccine (Butantan-DV) [9]. The study was conducted at a single site: *Centro Integrado de Ensaios Clínicos* (CIDEC) of Oswaldo Cruz Foundation, located in Simões Filho, a municipality within the Metropolitan Region of Salvador, Bahia state, Brazil.

The population consisted of children and adolescents aged 2–17 years-old at the time of study entry who are either healthy or have controlled comorbidities. The inclusion criteria were participation in the RCT and the availability of blood samples for this sub study at baseline. Cases that tested positive for IgG anti-CHIKV at baseline were excluded from the follow-up analysis. All patients were included between January 2018 and January 2020. The inclusion date for this sub study (baseline) was established as the first blood sample collection available for serological testing after six months of recruitment into the RCT.

Surveillance for arbovirus infection was conducted through three types of medical visits: routine, adverse event and fever visits. A complete description of the visits protocol was previously published [11]. Briefly, the routine visits were scheduled periodically (total of 10 for each participant of the RCT), to collect clinical data about any symptoms and blood samples. The adverse event visits occurred in case of symptoms or illnesses, and only clinical data was obtained. The fever visit occurred if participant reported fever, with collection of clinical data and blood samples for RT-PCR tests (CHIKV, DENV, and ZIKV) if sample were obtained within nine days of symptoms onset. During medical visits, a structured questionnaire was administered, which included specific questions about adverse events of special interest (fever, headache, exanthema, fatigue, arthralgia, myalgia, retroocular pain, diarrhea, abdominal pain, vomiting, conjunctivitis, and arthritis. No specific arbovirus prevention measures such as use of repellents or mosquito nets was provided for participants during this study and was not part of the original dengue vaccine trial protocol.

### Classification according to disease duration

To classify acute, subacute, and chronic chikungunya, we analyzed all clinical and laboratory data obtained from any medical visit performed up to 4 years of follow-up. To ensure the time of onset of symptoms, only cases that presented a positive RT-PCR for CHIKV were classified. The duration of osteoarticular and muscular symptoms/signs was used to define the cases as acute, subacute, or chronic: up to 14 days was classified as acute; from 14 days up to three months, subacute; and more than three months, chronic [10].

### Determination of symptomatology

Patients were considered symptomatic if they experienced at least one episode of the following symptoms/signs after excluding other possible concurrent diagnosis: fever, headache, myalgia, arthralgia, cutaneous rash, retro-ocular pain, arthritis, vomiting, abdominal pain, and diarrhea. By definition, all RT-PCR positive cases were symptomatic since fever was an inclusion criterion for molecular testing. For cases positive only by serology, the distinction between symptomatic and asymptomatic was based on the clinical data registered in the structured questionnaires during routine or adverse event visits throughout the follow-up period. If at least one of the following arbovirus symptoms were reported, the individual was considered symptomatic: fever, headache, exanthema, fatigue, arthralgia, myalgia, retroocular pain, diarrhea, abdominal pain, vomiting, conjunctivitis, and arthritis.

### RT-PCR and serologic tests

During fever visits, blood samples obtained within nine days after disease onset were tested for CHIKV, DENV, and ZIKV by RT-PCR tests performed at the Adolfo Lutz Laboratory following the methodology described by Lanciotti *et al* [11–13]. The results were provided with qualitative information displayed (positive and negative). For cases positive for CHIKV in RT-PCR, we conducted antibody measurements at two and three to four years of follow-up to assess the duration and characteristics of the humoral response.

The serological tests consisted of an enzyme-linked immunosorbent assay (ELISA, Euroimmun, EI 293a-9601) to detect anti-chikungunya IgG Neutralization Test (PRNT90).

ELISA was performed at baseline and in a median of 26 months (IQR = 22–30) after baseline. Seroconversion for chikungunya was defined as a positive test result with a ratio ≥ 1, following the test protocol. In individuals who tested positive by RT-PCR, a third serological test was performed three to four years after positive RT-PCR to determine the duration of humoral response. The 90% Plaque Reduction PRNT90 was conducted following a previously described [14] protocol, with minor adjustments. Briefly, PRNT90 was employed to identify the maximum serum dilution (1:20, 1:80, 1:320, and 1:1280) required to reduce arbovirus plaque formation by 90% in Vero cells, using a CHIKV strain isolated in Brazil. Titers ≥20 were considered positive.

### Statistical analysis

Descriptive analysis was performed using the software GraphPad Prism (version 10.0). Categorical variables were expressed in absolute values and percentages. Continuous variables with normal distribution were expressed in median and standard deviation. To calculate the incidence of chikungunya in Simões Filho, we used, as denominator, the pediatric population estimates from the Brazilian Institute of Geography and Statistics (Instituto Brasileiro de Geografia e Estatística - IBGE), stratified by age (1–19 years), and, as numerator, the number of chikungunya cases notified in the municipality, between January, 2018 and December, 2022 from the TabNet platform of the Unified Health System Department of Informatics (DATASUS- *Departamento de Informática do Sistema Único de Saúde do Brasil*, http://www2.datasus.gov.br). Incidence was expressed in cases per 1,000 inhabitants. The incidence of cases in the pediatric cohort was estimated using, as numerator, the number of positive RT-PCR cases and, as denominator, the cohort population, by year, and was expressed as cases per 1,000. The normality of the numerical variables was verified through descriptive statistics, graphical analysis, and the Kolmogorov-Smirnov test.

## Results

We excluded 23 (7%) individuals who were seropositive for CHIKV at baseline (Table A in S1 Text). Most participants (311/325-95.7%) had more than two years of follow-up. Cumulative incidence was 16%, resulting in a cumulative seropositivity (participants and excluded cases) of 22%.

We recruited 348 individuals for this study, consisting of 300 children 2–11 years old and 48 adolescents 12–17 years old. We excluded 23 (7%) individuals who were seropositive for CHIKV at baseline (Table A in S1 Text). Most participants (311/325-95.7%) had more than two years of follow-up. A total of 53 (16.3%) participants had a positive test for Positivity for CHIKV, with 25 cases detected by RT-PCR and 28 by serology (Fig A in S1 Text). PCR-positive cases were mainly detected from January to October 2020 (Fig B in S1 Text) when the incidence of chikungunya in Simões Filho was 3,4/1,000. Three participants in this sub study tested positive for DENV by RT-PCR during a period that did not coincide with the detection of CHIKV infection cases. No cases of ZIKV were detected during the follow-up period.

RT-PCR tests were carried out in a median of three days (IQR = 2-4.5) after symptom onset. All cases detected by RT-PCR presented with fever as this was a condition for molecular testing.

A median of three (IQR = 2–5) symptoms/signs were reported, including headache (n = 16, 64%), myalgia (n = 12, 48%), arthralgia (n = 10, 40%), rash (n = 10, 40%), fatigue (n = 10, 40%), retro ocular pain (n = 4, 16%) and gastrointestinal symptoms (n = 2, 8%) (Table 1).

**Table 1:** Clinical and sociodemographic characteristics of individuals who tested positive in RT-PCR, serology, and those who tested negative for Chikungunya during the follow-up period.

	Negative	Positive	Positive
	n = 258	RT-PCR n = 25 (%)	Serology n = 28 (%)
**Age in years at T1 (median IQR)**	5 (3–9)	9 (3–13)	7 (5–12)
**Female (%)**	134 (52)	12 (48)	18 (64)
**Follow-up time (median IQR)**	24 (22–30)	43 (41–53)	42 (39–45)
**Acute phase signs/symptoms**			
Fever	N/A	25 (100)	23 (82)
Headache	N/A	16 (64)	17 (61)
Arthalgia	N/A	10 (40)	8 (28)
Myalgia	N/A	12 (48)	13 (46)
Exanthema	N/A	10 (40)	7 (25)
Retroocular/orbital pain	N/A	4 (16)	3 (11)
Arthritis	N/A	2 (8)	2 (7)
Vomiting	N/A	2 (8)	1 (3)
Abdominal pain	N/A	1 (4)	3 (10)
Diarrhea	N/A	N/A	1 (3)

Data are presented as median (interquartile range 25–75%) and absolute numbers with (%).

N/A= Not applicable.

Among the 28 cases of chikungunya detected through serology, 15 (53.57%) were also confirmed by RT-PCR. Additionally, 16/28 (57.1%) reported fever accompanied by at least one of the following symptoms or signs commonly associated with arboviral infections: arthralgia, arthritis, myalgia, or rash (Table 1). The two most common symptoms or signs were fever (82%) and headache (61%) in children. Two (7.1%) reported at least one episode of fever during the follow-up period, with no additional manifestation, and five (17.8%) did not report symptoms/signs. The symptoms/signs listed were recorded in structured questionnaires in prospective medical assessments, and no positive RT-PCR test for the other arbovirus was detected in this group during the follow-up period (Fig C in S1 Text).

Five out of 53 individuals (9.4%) did not present symptoms during the follow-up period and were classified as asymptomatic. 44/53 (83.0%) individuals positive for chikungunya in RTPCR or serology were considered polysymptomatic, including 23 RT-PCR positives and 21 positives only in serologic tests. The remaining were classified as oligosymptomatic (n = 4, 7.5%), as they presented only one symptom (all reported fever).

To assess the duration of chikungunya symptoms in the pediatric population, we analyzed all required and auto-referred symptoms reported during scheduled and unscheduled visits after the date of the positive RT-PCR test. Most individuals (n = 21; 84%) recovered at day 4 (IQR = 24) after symptoms onset and were classified as acute chikungunya. One case classified as subacute presented persistent myalgia up to 40 days after the onset of symptoms, with no other symptom reported during the remaining 16 months of follow-up.

Three cases persisted with symptoms for more than three months and were classified as chronic chikungunya (12%) - with respective ages of 4, 11 and 13 years. These patients presented with joint pain and edema, dermatitis, and myalgia that continued for more than 11 months and reported functional limitations in basic daily activities due to persistent polyarthralgia (Table 2).

**Table 2:** Characteristics of participants with post-acute symptoms.

	Patient 01	Patient 02	Patient 03	Patient 04
**Classification**	Subacute	Chronic	Chronic	Chronic
N of visits	3	11	8	5
Signs/symptoms in the acute phase	2	4	2	3
Medication	NI	Paracetamol/ Dexamethasone/ Prednisone/ Ibuprofen/Teflan	NI	Prednisone
Location of Pain in the Chronic Phase	NI	Polyarticular and bilateral pattern	NI	Polyarticular
Functional limitation	NI	Difficulty in walking and performing daily activities	NI	Difficulty in walking and performing daily activities/household
Follow-up time in months	16	31	17	45
Duration of symptoms in months	1	16	11	28

Data presented in absolute numbers; NI – Not Informed.

NI – Not Informed.

**Fig 1:**
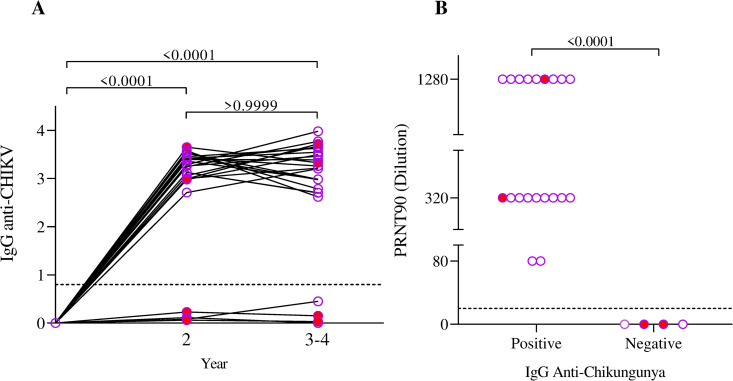
Humoral immune response and neutralizing antibody titers in pediatric CHIKV infection. **(A) **IgG Anti-Chikungunya titters in the positive PCR test at baseline, 2 and 3-4 years after the onset of the disease. The red dots represent patients with post-acute symptoms. **(B)** Comparison of PRNT90 (Dilution) titters between IgG Anti-Chikungunya positive and negative samples. Each point represents an individual sample. The red dots represent patients with post-acute symptoms.

We also analyzed the seroconversion rate and duration of specific IgG antibody titters among RT-PCR positive participants. Only 4/25 (16%) participants did not seroconvert in two tests performed up to 2 (IQR = 1,25–2) years after symptom onset. Non-seroconverts were under 6 years old and presented with one or two symptoms at acute phase (Table B in S1 Text). Two nonseroconverted cases persisted with symptoms for more than one month. On the other hand, the majority of participants with positive RT-PCR tests presented high anti-CHIKV IgG titers two years after disease (p < 0.0001) onset and persisted for up to four years (Fig 1). We further investigated the stability of IgG levels over time among seroconverted individuals. Serum from patients who did not seroconvert failed to neutralize the virus, whereas those from patients who seroconverted successfully neutralized it (*p* < 0.0001) (Fig 1).

## Discussion

Our study found a cumulative incidence of 16% for chikungunya and a cumulative seropositivity of 22% in the pediatric population. These findings are particularly notable given that the study was conducted in a region endemic to the chikungunya virus. The relatively low seropositivity aligns with previous studies showing that even after multiple outbreaks, a substantial proportion of adolescents and children remain seronegative and, consequently, at risk for future infections [17].

A high rate of susceptibility to CHIKV infection despite a long period of endemicity was reported in other studies. In Nicaragua, following the first CHIKV epidemic in 2015, the seroconversion rate for children ages 2–14 and ages 15 and older was 6.1% and 13.1%, respectively [15]. In another study in Kenya, an endemic region, 6.4% of children aged 7 years (IQR 5–10) seroconverted to CHIKV between 2014 and 2018 [16], reinforcing the majority of the population remaining at risk. The analysis of disease prevalence in a susceptible pediatric population helps understand the dynamics of chikungunya’s spread. Examining infectivity and its transmission rate is crucial for developing strategies to manage and mitigate their impact on public health and highlighting the relevance of disease control strategies and preventive measurements despite long-term CHIKV circulation in these areas.

Our study added value to the existing knowledge on the clinical presentation of chikungunya in children and adolescents. First, we reported that a small proportion of children presented asymptomatic/oligosymptomatic infection. The proportion of asymptomatic individuals varied from 7% (when considered as symptomatic individuals with at least one episode of fever during follow-up) to 32%, when criteria included other acute symptoms and a negative molecular test for other arboviruses. Although other studies have estimated up to 40% of asymptomatic infection in pediatric patients, this is the first study to prospectively monitor symptomatology in a cohort with active vigilance for arboviruses and routine tests for other arboviruses [8]. The employed methodology adds robustness to the findings that most children experience a symptomatic CHIKV infection. Investigating the rate of symptoms helps understand the disease’s hidden burden and plan effective strategies to control CHIKV spread. Considering that the majority (~85%) presented two or more symptoms and fever was reported by up to 93% in this period, surveillance of this symptom during CHIKV outbreaks may help early detection of new pediatric cases, allowing monitoring of the spread and implementing control measures. Similar clinical patterns were observed in a pediatric cohort in Rio de Janeiro during the 2019 outbreak, in which fever (90.2%) and arthralgia (76.5%) were the most frequent symptoms, reinforcing the similarity of chikungunya presentation in children and adults even in different endemic contexts [17].

Second, we estimated around 15% of post-acute symptoms among pediatric patients with confirmed infection by molecular tests. The frequency was slightly inferior compared to 2336% of pos-acute symptoms detected in a more extensive cohort study pediatric in Managua (Nicaragua) [7]. The age of the pediatric population included in this study (median, 11 yearsold) and ours (median, 5 years-old) may explain this difference since the risk of chronicity seems to increase with age. Similarly, in a pediatric subanalysis of a Colombian cohort, the prevalence of post-chikungunya arthralgia increased progressively with age: from 24.1% in children under 10 years to 35.5% in adolescents aged 15–19 years [21]. This age-related trend further reinforces the hypothesis that older children and adolescents are more susceptible to long-term outcomes associated with CHIKV infection [18,19]. Although lower compared to 60% in adults in this region, the frequency of chronic arthralgia is not neglected and will aggravate the social and economic impact in affected areas [2]. Persistent polyarthralgia in these patients led to difficulties in performing even light activities such as household tasks, simple physical exercises, and school responsibilities significantly disrupting routines and quality of life.

We observed sustained persistence and neutralizing activity of high IgG titers against CHIKV in all children who seroconverted two years after a positive RT-PCR result. Notably, there was no evidence of a significant decline in IgG levels in most participants over the follow-up period.

Remarkably, 16% of the children did not seroconvert despite confirmed RT-PCR infections. This observation was corroborated by a follow-up serological test conducted one year later and a neutralization assay. These findings have important implications for serological surveys, as they may underestimate the true prevalence of CHIKV infections in pediatric populations. The lack of seroconversion or neutralizing activity in young children after CHIKV infection highlights critical considerations for vaccine development. It is possible that PCR-positive participants that did not seroconvert have developed T-cell immune memory despite the absence of detectable antibody response. Further studies should be performed to evaluate anti- CHIKV cell response in the absence of a humoral response. Furthermore, these findings underscore the potential need for vaccination strategies in pediatric populations, as a significant proportion of children may not achieve full immunity following natural infection, possibly leaving them at risk for future CHIKV infections. Given the results of previous studies showing successful seroconversion and neutralizing antibody response in adults after vaccination [20] further research into vaccine efficacy in children is essential to ensure a comprehensive protection for this vulnerable group.

The major strength of this study is the active surveillance of the study population and regular molecular testing for each febrile episode necessary to determine incidence, asymptomatic rate and disease duration. However, our study has several limitations. First, the low number of chronic cases did not allow us to evaluate risk factors among children. Second, the frequency of asymptomatic cases can be overestimated. Despite the comprehensive surveillance approach, a portion of CHIKV infections was not detected during the acute phase, likely due to the larger CHIKV outbreak coinciding with the COVID-19 pandemic, which may have influenced healthcare-seeking behavior. Additionally, the absence of RT-PCR screening for non-febrile individuals may have resulted in undetected asymptomatic infections in cases that failed to seroconvert (IgG–/RT-PCR+), potentially underestimating the true burden of asymptomatic chikungunya. Third, the population recruited for this study was enrolled in a dengue vaccine clinical trial. Exclusion criteria such as chronic or immunosuppressive conditions and the use of immunosuppressive therapies may have affected the external representativeness of the immunological findings. However, these conditions are uncommon in children, affecting only 2.6% of this population [22]. Therefore, the results of this study reflect a population of healthy children and adolescents without comorbidities or immunosuppression. To assess whether dengue vaccination could have influenced susceptibility to CHIKV infection, we compared the proportions of CHIKV-positive and CHIKV-negative individuals between the placebo and vaccinated groups. No significant differences (p = 0,7510) were found, indicating that dengue vaccination did not affect the risk of CHIKV infection in this pediatric cohort. This finding supports the generalizability of our results regarding CHIKV infection dynamics, independent of dengue vaccine status (Fig D in S1 Text). In summary, this study provided important information on the natural history of chikungunya in the pediatric population, such as the low chronicity rate, a significant frequency of nonseroconversions and the existence of long-lasting immunity post-exposure after seroconversion. Determining the dynamics of chikungunya infection in a pediatric population helps plan healthcare resources, including hospital beds, pediatric care, and rehabilitation services, during and after outbreaks. It can assist in developing prevention and control strategies for the disease.

## Supporting information

S1 Text **Fig A**. Flowchart showing the pediatric population included in the cohort and their classification according to laboratory tests. **Fig B**. Incidence of Chikungunya by year according to reported cases in the general population of the municipality of Simões Filho (blue line) and in the pediatric cohort (orange line). **Fig C. (A)** UpSet plot showing the frequency of symptoms among symptomatic patients who tested positive for Chikungunya by either serology or RT-PCR (n = 48). **(B)** UpSet plot showing the frequency of symptoms among patients who tested positive by RT-PCR only (n = 25). **(C)** UpSet plot showing the frequency of symptoms among patients who tested positive by serology only (n = 23). Note: Five asymptomatic patients were not included in the UpSet plots. **Fig D.** Proportion of Chikungunya virus (CHIKV) positive and negative individuals in the placebo and dengue-vaccinated groups in the pediatric cohort. In the placebo group, 18% (n = 19/105, orange bars) were CHIKV-positive and 82% (n = 86/105, blue bars) were CHIKV-negative. In the vaccinated group, 16.5% (n = 34/206, orange bars) were CHIKV-positive and 83.5% (n = 172/206, blue bars) were CHIKV-negative. Absolute numbers are shown above each bar. (Fisher’s exact test, p = 0.75). **Table A.** Sociodemographic characteristics of individuals who were seropositive and seronegative at baseline. Data are presented as median (interquartile range 25–75%) and absolute numbers with percentages (%). N/A = Not applicable. **Table B.** Characteristics of seronegative patients who later tested positive by RT-PCR.
(DOCX)

## References

[1] Kang H, Auzenbergs M, Clapham H, Maure C, Kim J-H, Salje H, et al. Chikungunya seroprevalence, force of infection, and prevalence of chronic disability after infection in endemic and epidemic settings: a systematic review, meta-analysis, and modelling study. Lancet Infect Dis. 2024;24(5):488–503. doi: 10.1016/S1473-3099(23)00810-1 38342105

[2] de Moraes L, Cerqueira-Silva T, Nobrega V, Akrami K, Santos LA, Orge C, et al. A clinical scoring system to predict long-term arthralgia in Chikungunya disease: A cohort study. PLoS Negl Trop Dis. 2020 Jul 21;14(7):e0008467. 10.1371/journal.pntd.0008467 PMC7373495 32693402

[3] Sissoko D, Malvy D, Ezzedine K, Renault P, Moscetti F, Ledrans M, et al. Post-epidemic Chikungunya disease on Reunion Island: course of rheumatic manifestations and associated factors over a 15-month period. PLoS Negl Trop Dis. 2009;3(3):e389. doi: 10.1371/journal.pntd.0000389 19274071 PMC2647734

[4] Tjaden NB, Suk JE, Fischer D, Thomas SM, Beierkuhnlein C, Semenza JC. Modelling the effects of global climate change on Chikungunya transmission in the 21st century. Sci Rep. 2017;7(1):3813. doi: 10.1038/s41598-017-04012-5 28630444 PMC5476675

[5] Cerqueira-Silva T, Pescarini JM, Cardim LL, Leyrat C, Whitaker H, Antunes de Brito CA. Risk of death following chikungunya virus disease in the 100 million Brazilian cohort, 2015–18: a matched cohort study and self-controlled case series. Lancet Infect Dis. 2024;24(5):504–13. 38342106 10.1016/S1473-3099(23)00739-9

[6] Sissoko D, Moendandze A, Malvy D, Giry C, Ezzedine K, Solet JL. Seroprevalence and risk factors of chikungunya virus infection in Mayotte, Indian Ocean, 2005-2006: a population-based survey. PLoS One. 2008;3(8):e3066. doi: 10.1371/journal.pone.0003066 PMC2518850 18725980

[7] Warnes CM, Bustos Carrillo FA, Zambrana JV, Lopez Mercado B, Arguello S, Ampié O, et al. Longitudinal analysis of post-acute chikungunya-associated arthralgia in children and adults: A prospective cohort study in Managua, Nicaragua (2014-2018). PLoS Negl Trop Dis. 2024;18(2):e0011948. doi: 10.1371/journal.pntd.0011948 38416797 PMC10962812

[8] Ritz N, Hufnagel M, Gérardin P. Chikungunya in Children. Pediatr Infect Dis J. 2015;34(7):789–91. doi: 10.1097/INF.0000000000000716 26069950

[9] Kallás EG, Cintra MAT, Moreira JA, Patiño EG, Braga PE, Tenório JCV. Live, attenuated, tetravalent butantan–dengue vaccine in children and adults. New England Journal of Medicine. 2024;390(5):397–408. 38294972 10.1056/NEJMoa2301790

[10] Brasil. Chikungunya manejo clínico. 2017.

[11] Lanciotti RS, Kosoy OL, Laven JJ, Panella AJ, Velez JO, Lambert AJ. Chikungunya virus in US travelers returning from India, 2006. Emerg Infect Dis. 2007;13(5):764–7. 17553261 10.3201/eid1305.070015 PMC2738459

[12] Johnson BW, Russell BJ, Lanciotti RS. Serotype-specific detection of dengue viruses in a fourplex real-time reverse transcriptase PCR assay. J Clin Microbiol. 2005;43(10):4977–83. doi: 10.1128/JCM.43.10.4977-4983.2005 16207951 PMC1248506

[13] Lanciotti RS, Kosoy OL, Laven JJ, Velez JO, Lambert AJ, Johnson AJ, et al. Genetic and serologic properties of Zika virus associated with an epidemic, Yap State, Micronesia, 2007. Emerg Infect Dis. 2008;14(8):1232–9. doi: 10.3201/eid1408.080287 18680646 PMC2600394

[14] Baer A, Kehn-Hall K. Viral concentration determination through plaque assays: using traditional and novel overlay systems. Journal of Visualized Experiments. 2014;93. 10.3791/52065 PMC4255882 25407402

[15] Kuan G, Ramirez S, Gresh L, Ojeda S, Melendez M, Sanchez N. Seroprevalence of anti-chikungunya virus antibodies in children and adults in managua, nicaragua, after the first Chikungunya epidemic, 2014-2015. PLoS Negl Trop Dis. 2016;10(6). 10.1371/journal.pntd.0004773 PMC4913910 27322692

[16] Khan A, Bisanzio D, Mutuku F, Ndenga B, Grossi-Soyster EN, Jembe Z, et al. Spatiotemporal overlapping of dengue, chikungunya, and malaria infections in children in Kenya. BMC Infect Dis. 2023;23(1):183. doi: 10.1186/s12879-023-08157-4 36991340 PMC10053720

[17] Gomes PD, Carvalho RFSM, Massini MM, Garzon RH, Schiavo PL, Fernandes RC de SC. High prevalence of arthralgia among infants with Chikungunya disease during the 2019 outbreak in northern region of the state of Rio de Janeiro. Front Pediatr. 2022;10. doi: 10.3389/fped.2022.00000 PMC9627548 36340716

[18] Simon F, Caumes E, Jelinek T, Lopez-Velez R, Steffen R, Chen LH. Chikungunya: risks for travellers. J Travel Med. 2023;30(2). 10.1093/jtm/taad008 PMC10075059 36648431

[19] Kuan G, Ramirez S, Gresh L, Ojeda S, Melendez M, Sanchez N. Seroprevalence of Anti-Chikungunya virus antibodies in children and adults in managua, nicaragua, after the first Chikungunya epidemic, 2014-2015. PLoS Negl Trop Dis. 2016;10(6). 10.1371/journal.pntd.0004773 PMC4913910 27322692

[20] Schneider M, Narciso-Abraham M, Hadl S, McMahon R, Toepfer S, Fuchs U, et al. Safety and immunogenicity of a single-shot live-attenuated chikungunya vaccine: a double-blind, multicentre, randomised, placebo-controlled, phase 3 trial. Lancet. 2023;401(10394):2138–47. doi: 10.1016/S0140-6736(23)00641-4 37321235 PMC10314240

[21] Rodriguez-Morales AJ, Gil-Restrepo AF, Ramírez-Jaramillo V, Montoya-Arias CP, Acevedo-Mendoza WF, Bedoya-Arias JE, et al. Post-chikungunya chronic inflammatory rheumatism: results from a retrospective follow-up study of 283 adult and child cases in La Virginia, Risaralda, Colombia. F1000Res. 2016;5:360. doi: 10.12688/f1000research.8235.2 27081477 PMC4813633

[22] Patel M, Chen J, Kim S, Garg S, Flannery B, Haddadin Z, et al. Analysis of marketscan data for immunosuppressive conditions and hospitalizations for acute respiratory illness, United States. Emerg Infect Dis. 2020;26(8):1720–30. doi: 10.3201/eid2608.191493 32348234 PMC7392442

