# Supplementary material for: Chikungunya in a pediatric cohort: Asymptomatic infection, seroconversion, and chronicity rates
Source: PLoS Negl Trop Dis. 2025 Jul 16;19(7):e0013254. doi: 10.1371/journal.pntd.0013254 (PMC12286324; doi:10.1371/journal.pntd.0013254)
Supplement: S1 Text — Fig A. Flowchart showing the pediatric population included in the cohort and their classification according to laboratory tests. Fig B. Incidence of Chikungunya by year according to reported cases in the general population of the municipality of Simões Filho (blue line) and in the pediatric cohort (orange line). Fig C. (A) UpSet plot showing the frequency of symptoms among symptomatic patients who tested positive for Chikungunya by either serology or RT-PCR (n = 48). (B) UpSet plot showing the frequency of symptoms among patients who tested positive by RT-PCR only (n = 25). (C) UpSet plot showing the frequency of symptoms among patients who tested positive by serology only (n = 23). Note: Five asymptomatic patients were not included in the UpSet plots. Fig D. Proportion of Chikungunya virus (CHIKV) positive and negative individuals in the placebo and dengue-vaccinated groups in the pediatric cohort. In the placebo group, 18% (n = 19/105, orange bars) were CHIKV-positive and 82% (n = 86/105, blue bars) were CHIKV-negative. In the vaccinated group, 16.5% (n = 34/206, orange bars) were CHIKV-positive and 83.5% (n = 172/206, blue bars) were CHIKV-negative. Absolute numbers are shown above each bar. (Fisher’s exact test, p = 0.75). Table A. Sociodemographic characteristics of individuals who were seropositive and seronegative at baseline. Data are presented as median (interquartile range 25–75%) and absolute numbers with percentages (%). N/A = Not applicable. Table B. Characteristics of seronegative patients who later tested positive by RT-PCR. (DOCX) [file pntd.0013254.s001.docx]

This supplementary file provides additional figures and tables that complement and support the study’s main results.

Fig A in S1 Text

Flowchart with pediatric population included in the cohort and the classification according to laboratory exams.


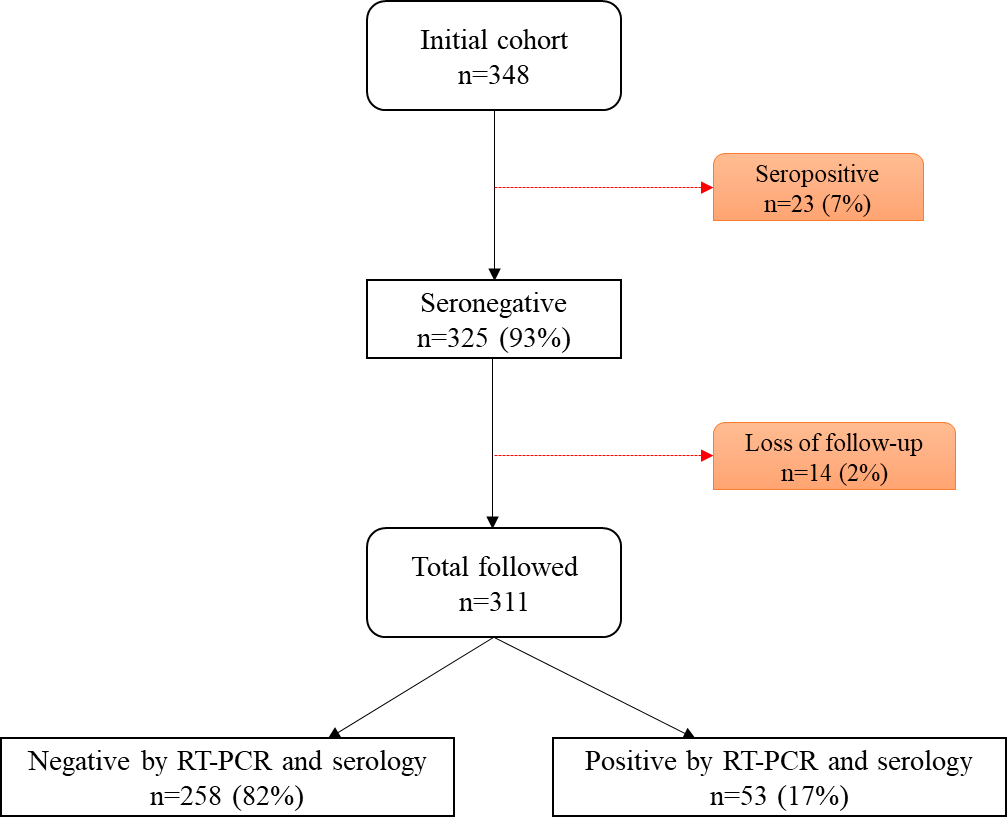


Fig B in S1 Text

Incidence of Chikungunya by year according to the notification in the population of the municipality of Simões Filho (blue line) and in the population of the pediatric cohort (orange line).


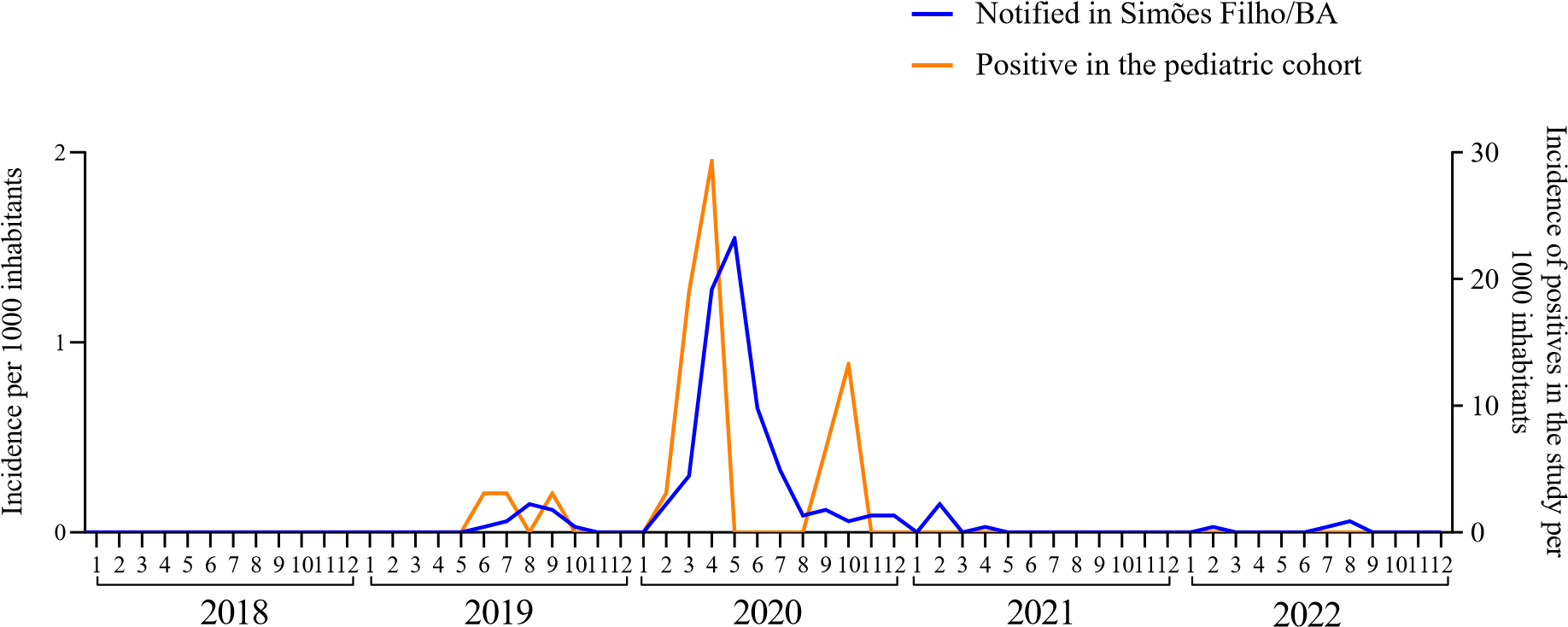


Fig C in S1 Text

UpSet plots showing the frequency of symptoms among symptomatic patients who tested positive for Chikungunya via serology or RT-PCR (n=48) (A), tested positive via RT-PCR (n=25) (B), or tested positive via serology (n=23) (C). Five asymptomatic patients were not included in the UpSet plot.

A


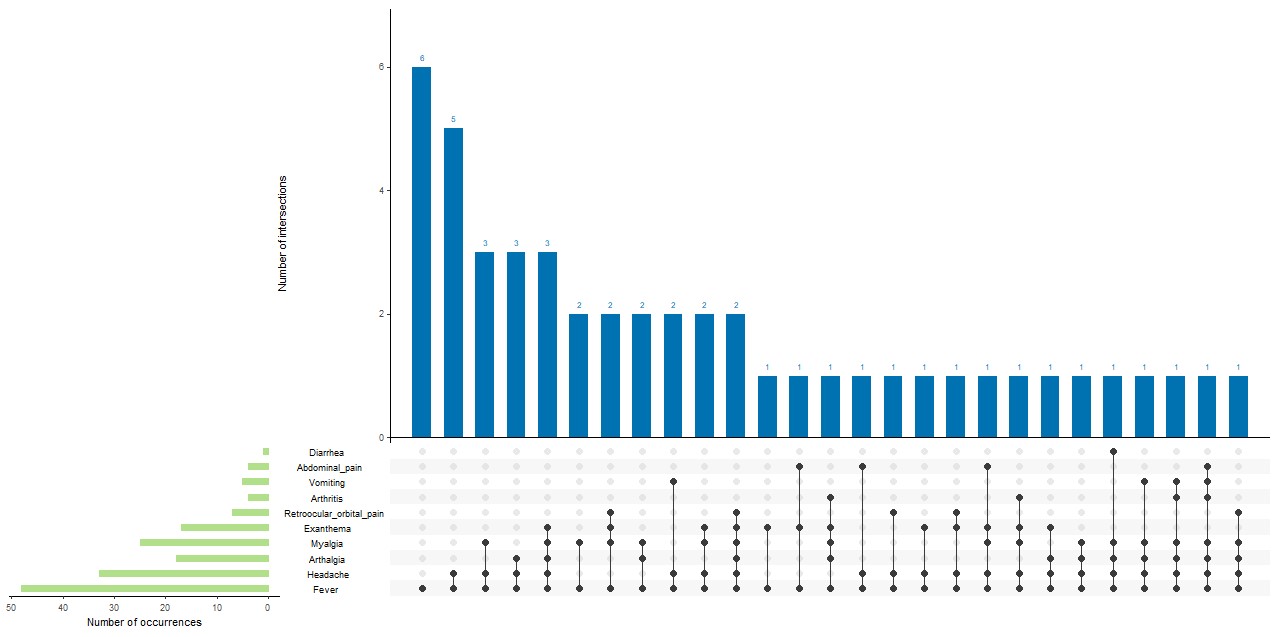


B


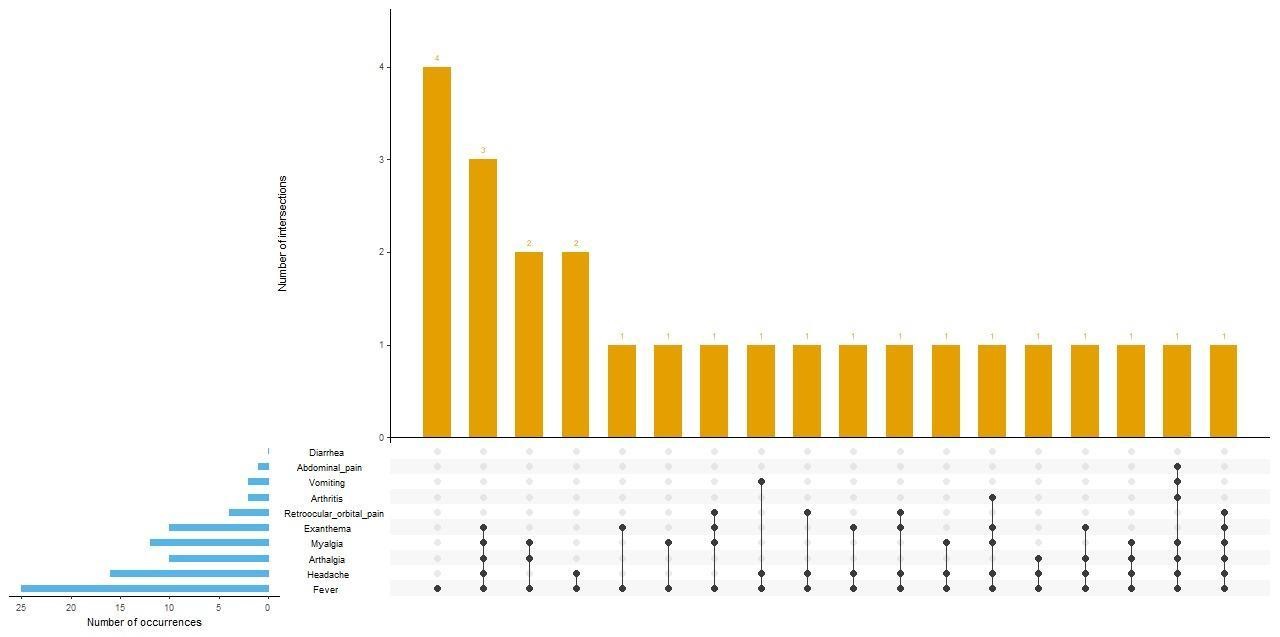


C


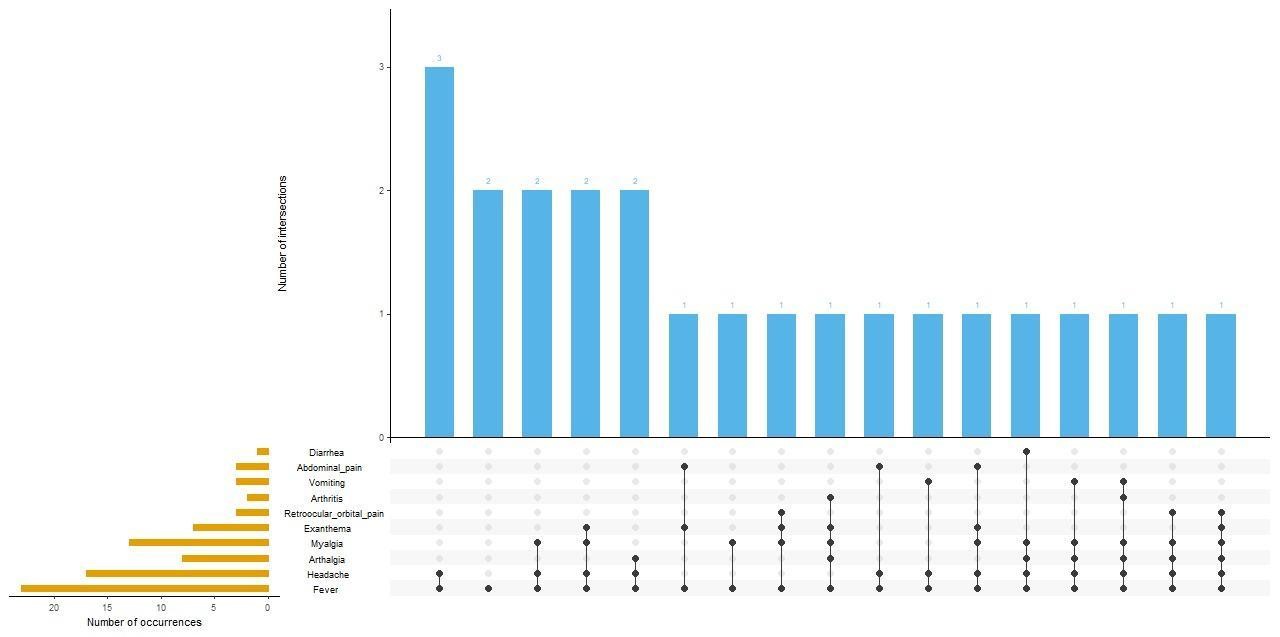


Fig D in S1 Text

Proportion of Chikungunya virus (CHIKV) positive and negative individuals in the placebo and dengue-vaccinated groups in the pediatric cohort. In the placebo group, 18% (n = 19/105, orange bars) were CHIKV-positive, while 82% (n = 86/105, blue bars) were CHIKV-negative. In the vaccinated group, 16.5% (n = 34/206, orange bars) were CHIKV-positive, and 83.5% (n = 172/206, blue bars) were CHIKV-negative. Absolute numbers are shown at the top of each bar. (Fisher’s exact test, *p* = 0.75).

Placebo

DENV

0

50

100

%

o

f

p

a

r

t

i

c

i

p

a

n

t

s

Vaccinated

CHIKV Negative

CHIKV Positive

Table A in S1 Text

Sociodemographic characteristics of individuals seropositive and seronegative at baseline. Data are presented as median (interquartile range 2575%) and absolute numbers with (%). N/A= Not applicable

|  | Total n=348 (%) | Soropositive n=23 (7%) | Soronegative n=325 (93%) |
| --- | --- | --- | --- |
| Age in years at T1 (median IQR) | 6 (3-10) | 7,5 (5-11) | 5 (2-10) |
| Female | 182 (52) | 12 (52) | 170 (52) |
| Race/color  Multiracial | 215 (62) | 11 (48) | 204 (63) |
| White | 35 (10) | 1 (4) | 34 (10) |
| Black | 94 (27) | 9 (39) | 85 (26) |
| Indigenous | 1 (0.3) | N/A | 1 (0.4) |
| Asian | 3 (0.7) | 1 (4) | 2 (0.6) |

Table B in S1 Text.

Characteristics of seronegative patients after positive RT-PCR

| Patient 01 | | Patient 02 | Patient 03 | Patient 04 |
| --- | --- | --- | --- | --- |
| Classification | Acute | Acute | Subacute | Chronic |
| Age (RT-PCR) | 3 | 5 | 6 | 4 |
| Sex | Female | Female | Female | Female |
| Duration of illness at Days post symptoms onset at RT-PCR | 4 | 3 | 6 | 3 |
| Signs/symptoms in the acute phase | Fever | Fever/Myalgia | Fever/Headache Fever/Headache | |
